# The Mediating Role of Blood Metabolites in the Association between Basal Metabolic Rate and Obstetrical Disorders: A Mendelian Randomization Analysis

**DOI:** 10.2174/0118715303400445250718112316

**Published:** 2025-07-24

**Authors:** Yanqiong Gan, Xinlin Tan, Yu Tang, Qi Shi, Hongbo Qi

**Affiliations:** 1 Department of Obstetrics, The First Affiliated Hospital of Chongqing Medical University, No.1, Youyi Road, Chongqing 400016, China;; 2 Department of Obstetrics, The Affiliated Hospital of North Sichuan Medical College, No.63, Wenhua Road, Nanchong, 637000, China;; 3 Chongqing Key Laboratory of Maternal and Fetal Medicine, Chongqing Medical University, Chongqing 400016, China;; 4 Joint International Research Laboratory of Reproduction and Development of Chinese Ministry of Education, Chongqing Medical University, 400016, China

**Keywords:** Basal metabolic rate, blood metabolite, intrahepatic cholestasis of pregnancy, mannose levels, Mendelian randomization, poor fetal growth

## Abstract

**Introduction:**

Previous studies suggest a link between Basal Metabolic Rate (BMR) and obstetrical disorders; however, causality remains unclear. We investigated the causal effects of BMR on 14 obstetric disorders and evaluated the potential mediating effects of blood metabolites in these relationships.

**Methods:**

Using Genome-Wide Association Study (GWAS) summary data, we conducted both univariate and multivariable Mendelian Randomization (MVMR) analyses. The primary causal inference was based on Inverse Variance Weighted (IVW), MR-Egger, weighted median, and sensitivity analyses (Cochran’s Q, MR-PRESSO). Mediation analysis was employed to quantify the proportion of effects operating through metabolite-regulated pathways.

**Results:**

BMR was inversely associated with hyperemesis gravidarum (OR=0.73, 95% CI: 0.59-0.90, *P*=0.008), Intrahepatic Cholestasis of Pregnancy (ICP) (OR=0.67, 95% CI: 0.56-0.80, *P*<0.001), poor fetal growth (OR=0.80, 95% CI:0.71-0.90, *P*=0.001), and preterm delivery (OR=0.78, 95% CI:0.70-0.87, *P*<0.001). MVMR identified elevated BMR and mannose levels as protective against ICP, with BMR showing a positive correlation with mannose. Mediation analysis revealed that BMR reduced ICP risk partly through increased mannose (OR = 1.38, 95% CI: 1.19-1.59, *P* = 2.03 × 10^−5^), accounting for 29.93% of the effect.

**Discussion:**

Elevated BMR significantly reduced risks of intrahepatic cholestasis (HR=0.67), fetal distress (HR=0.80), and preterm birth (HR=0.78), mediated partly by mannose levels. Mendelian randomization established causality, linking metabolic adaptation to improved pregnancy outcomes. However, these findings, based on European genetic data, limit generalizability, and unmeasured confounders may persist despite MR methods.

**Conclusion:**

Higher BMR may lower risks of hyperemesis gravidarum, ICP, poor fetal growth, and preterm delivery. Mannose mediates the protective effect of BMR on ICP, highlighting potential metabolic pathways for intervention.

## 1 INTRODUCTION

During pregnancy, the maternal body undergoes a series of complex physiological alterations to facilitate fetal development. Although these alterations are typical, they may also result in pathological conditions, obstetrical disorders, and adverse pregnancy outcomes, including gestational hypertension, gestational diabetes (GDM), miscarriage, preterm delivery, and low birth weight [1-5]. Obstetrical disorders significantly affect the health of mothers as well as infants [6]. Research indicates that the incidence and advancement of obstetrical disorders are influenced by various factors, including the mother's age, nutritional state, hormonal imbalances, immunological function, and genetic susceptibility [7]. Nonetheless, there is a lack of consistency among studies. The precise pathophysiology of obstetrical disorders remains ambiguous and requires validation through additional research.

Recently, studies have established a correlation between BMR and certain obstetrical disorders. The research examined metabolic alterations during pregnancy in women with metabolic diseases that could impact fetal development and the mother’s health by affecting glucose and lipid metabolism [8]. Studies also indicated that BMR is significantly correlated with insulin resistance, weight gain, and gestational hypertensive disorders [9, 10], which suggests the potential relationship between BMR and obstetrical disorders. In addition, the conclusions of several studies regarding the correlation between the two remain contentious due to differences in ethnic populations, geographic situations, and experimental techniques. The observed association is likely confounded by covariates, such as Body Mass Index (BMI) and metabolite profiles, rather than reflecting a direct causal effect of alterations in Basal Metabolic Rate (BMR) [11, 12].

Metabolites are the core molecules of energy metabolism and physiological regulation, and the changes in their levels can reflect the dynamic balance of the maternal metabolic state. BMR, which quantifies resting energy expenditure, serves as an integrated biomarker reflecting the systemic regulation of macronutrient homeostasis, particularly in carbohydrate metabolism, lipid oxidation, and protein turnover [13]. During pregnancy, metabolic demands increase, and placental function depends on the adequate supply of maternal metabolites (such as glucose and fatty acids) [14]. Recent studies have shown that the association between BMR and obstetric diseases may be mediated through a complex network involving metabolites. Within gestational metabolic networks, blood metabolites function as bioactive mediators. Their dual roles as real-time biomarkers of maternal-fetal metabolic interplay and as functional modulators (influencing placental nutrient transport, bile acid enterohepatic cycling, and macrophage polarization dynamics) collectively determine pregnancy viability. Metabolic imbalance may induce obstetric diseases (such as ICP, GDM, pre-eclampsia) through mechanisms such as oxidative stress, inflammation, or hormonal imbalance [15-18]. Elevated BMR may enhance liver glycogenolysis and fatty acid oxidation, affect the circulation levels of key metabolites (such as mannose), and regulate bile acid metabolism and placental function [19].

However, the association and mechanism between BMR and obstetrical disorders remain undetermined. Because observational studies may be influenced by confounding variables, such as genetic predisposition and environmental factors, establishing a causal relationship between the two is necessary [20]. Mendelian Randomization (MR) is an analytical method that uses genetic variations to deduce the bias between exposure factors and clinical outcomes [21]. Genetic diversity is randomly distributed and fixed at birth, remaining unaffected by environmental or social factors [22]. Mediation analysis examines the indirect pathways by which exposure factors affect outcomes through mediating variables. Integrating mediation analysis with MR enables the investigation of the potential causal correlation between an exposure factor and an illness outcome at the genetic level, while also elucidating the mediating variables and mechanisms underlying the causal effect.

Therefore, this study conducted a univariate MR analysis to investigate the potential causal correlation between BMR and obstetrical disorders, and further evaluated whether blood metabolites mediate the influence of BMR on the risk of developing obstetrical disorders through mediation analysis, thereby enhancing the understanding of the impact of genetic variants on individual traits and disease susceptibility. We found that BMR had an established relationship with hyperemesis gravidarum, ICP, poor fetal growth, and preterm delivery, and BMR may influence ICP through mannose.

## 2 MATERIALS AND METHODS

### 2.1 Study Design

This study investigated the causal effects of BMR on 14 obstetric disorders and evaluated the mediating roles of blood metabolites. The data concerning the pertinent phenotypes were sourced from published GWAS that have secured ethical approvals from their respective institutions. This paper utilizes solely publicly available data from these studies, obviating the necessity for further ethical approvals. This study followed the guidelines in strengthening the Reporting of Observational Studies in Epidemiology Using Mendelian Randomization (STROBE-MR). Initially, we evaluated the instrumental variables (IVs) for BMR and blood metabolites. Subsequently, we examined the causal correlation between BMR and obstetrical disorders in the two databases (FinnGen and UKBB). Finally, we conducted a meta-analysis to integrate the results from the two databases. Multivariable Mendelian randomization (MVMR) was first implemented to isolate BMR's direct effects, followed by causal mediation analysis quantifying metabolite-specific indirect effects on obstetric outcomes. This analysis identified BMR as the exposure factor, 14 obstetrical disorders as the outcome factors, and blood metabolites as the mediating factors. The MR analysis relies on three fundamental assumptions: association, requiring the instrumental variable to exhibit a strong correlation with exposure; exclusivity, which dictates that the instrumental variable is uncorrelated with confounders; and independence, positing that the instrumental variable is not correlated with the outcome [23].

### 2.2 Selection of Genetic Instrumental Variables

We detected Single-Nucleotide Polymorphisms (SNPs) that were strongly correlated (*P* < 5.0 × 10^ (-8)), had an F-statistic greater than 10, and had an r^2^ of 0.001 with BMR and 1400 blood metabolite expression as instrumental factors [24]. The SNPs selected in this study are all based on the published genome-wide association studies of BMR and blood metabolites, and the functional significance of these SNPs has been verified in multiple studies [25-28]. All SNPs' biological function annotations through the NHGRI-EBI GWAS Catalog (https://www.ebi.ac.uk/gwas/) and FUMA (http://fuma.ctglab.nl/) ensure their pathophysiological association with exposure (BMR) and mediating variables (blood metabolites). The *F*-statistic is derived from the variance explained (*R^2^*) by SNPs for each exposure by ((*R^2^*/(1−*R^2^*))×((*N*−*K*−1)/*K*)), where *K* is the number of SNPs and *N* represents the sample size of the GWAS study about these exposure factors. This study computed *R^2^* utilizing the Steiger filtering function from the Two-Sample MR in the R software. Ultimately, considering the potential multidirectional effects of the genetic instrument on possible confounders, we excluded SNPs from this analysis by utilizing the ldlink database (https://ldlink.nih.gov/) to eliminate multidirectional SNPs linked to other potential confounders [29, 30]. The loci with Linkage Disequilibrium (LD) associated with known confounding factors (such as BMI and diabetes-related SNPs) were excluded through the "SNP clip" function.

### 2.3 Data Sources

In this study, the genetic origin of participants was limited to Europeans to mitigate any potential bias arising from other ethnicities. GWAS data for obstetrical disorders were respectively obtained from the FinnGen consortium (FinnGen consortium, https://www.finngen.fi/en) [31] and UKBiobank (UKBiobank HRC-imputed, http://www.nealelab.is /uk-biobank/) [32]. Finally, we gathered GWAS summary-level data for 14 obstetrical disorders: hyperemesis gravidarum, hydatidiform mole, spontaneous abortion, premature rupture of membranes, poor fetal growth, delivery complicated with fetal distress, preterm delivery, gestational diabetes, gestational hypertension, pre-eclampsia or eclampsia, intrahepatic cholestasis of pregnancy, single spontaneous delivery, prolonged pregnancy, and postpartum depression. However, the UK Biobank lacks data on ICP and poor fetal growth. The sample sizes and information on these diseases in the two databases were presented in Table **1**.

### 2.4 Statistical Methods

The study utilized R software version 4.3.1, with primary packages including “Two-Sample MR” (version 0.5.7) and “MRPRESSO” (version 1.0) [33]. The correlation between BMR and the probability of each obstetrical disorder was presented as Odds Ratios (ORs) alongside 95% Confidence Intervals (CIs), with differences deemed statistically significant at *P* < 0.05 [34].

### 2.5 Univariable MR Analysis

The study primarily utilized two-sample MR to evaluate the causal connection between BMR and the 14 obstetrical disorders. The random effects model was employed to account for heterogeneity. When the random effects model was unavailable, the fixed effects model was used. This study also performed supplemental analyses utilizing MR-Egger regression, weighted median, weighted model, and simple model to further corroborate the dependability of the Inverse Variance Weighted (IVW) approach results. In this study, we employed the Benjamini-Hochberg technique to calculate the adjusted *p*-value and False Discovery Rate (FDR), aiming to minimize false-positive outcomes. In the analysis, the FDR of less than 5% was deemed statistically significant [35]. To enhance the reliability of our conclusions, we conducted a random-effects meta-analysis, integrating causality estimates from two distinct sources (FinnGen and UKBB).

### 2.6 Multivariable MR Analysis

In this study, the potential effect of blood metabolites as mediators of BMR and obstetrical disorders was investigated using MVMR and mediation analysis, and the proportion of mediating impacts was quantified [36, 37]. The mediation analysis proceeded in distinct steps: Path A (exposure to mediation) estimated the causal effect of BMR on metabolites (β-A) using two-sample MR. Path B (mediator to outcome) then estimated the causal effect of metabolites on obstetric diseases (β-B) by IVW. Finally, Total effect C' (exposure to outcome) represented the direct effect of BMR on the outcome after controlling for mediating variables. The mediating effect ratio was calculated as (β-A×β-B)/C', and the 95% confidence interval was estimated using the Bootstrap method (1000 iterations). All analyses are implemented through the R package "mediation" (version 4.5.0), and the code is sourced from GitHub (sample link) [38].

### 2.7 Sensitivity Analysis

Sensitivity analysis comprises a heterogeneity test, a horizontal pleiotropy test, and a reject-by-exclusion test. Initially, Cochran's Q test was employed to identify the heterogeneity among all SNPs [39]. Then, horizontal pleiotropy and the MR-Egger intercept test were used to detect the pleiotropic effect [40]. The MR-PRESSO program as also utilized to eliminate any outliers detected in the MR analysis and re-evaluated the MR causality estimations [41]. Following the removal of outliers, we assessed the robustness of the findings using a random effects model. The impact of specific SNPs on the overall causal estimates was confirmed using a case-by-case analysis [42]. In addition, we conducted the MR Steiger orientation analysis to validate the causal relationship between BMR and obstetrical disorders.

## 3 RESULTS

### 3.1 Instrumental Variable Characterization Parameters

In the univariate Mendelian randomization framework, genetic instruments were selected through a three-stage filtering process: (1) Genome-wide significance: SNPs attaining *P* < 5×10^−8^ in BMR GWAS; (2) LD clumping: Independent variants (r^2^ < 0.001, 10,000kb window) using 1000 Genomes European reference; (3) Strength validation: F-statistic >10 calculated as (β/SE)^2^ for each SNP. 6603 IVs were identified, all of which demonstrated robust associations with exposure. The pertinent parameters of the SNPs are outlined in Supplementary Table **1**.

### 3.2 Causal Effect of BMR and Obstetrical Disorders in Univariable MR

**Fig. (1):**
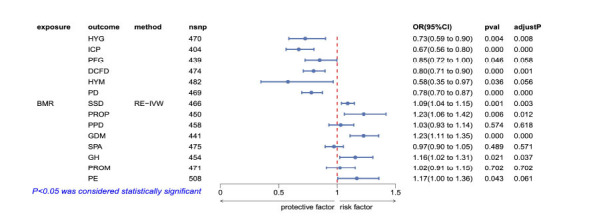
Mendelian randomization estimates BMR on the risk for 14 obstetrical diseases. “OR” represents the effect of a standard deviation increase in genetically predicted BMR on the odds of each outcome. **Abbreviations:** CI, Confidence Interval; OR, Odds Ratio; BMR, Basal Metabolic Rate; HYG, hyperemesis gravidarum; ICP, Intrahepatic Cholestasis of Pregnancy; PFG, Poor Fetal Growth; DCFD, Delivery Complicated with Fetal Distress; HYM, hydatidiform mole; PD, Preterm Delivery; SSD, Single Spontaneous Delivery; PROP, prolonged pregnancy; PPD, postpartum depression; GDM, gestational diabetes; SPA, spontaneous abortion; GH, Gestational Hypertension; PROM, Premature Rupture Of Membranes; PE, Pre-Eclampsia.

**Fig. (2):**
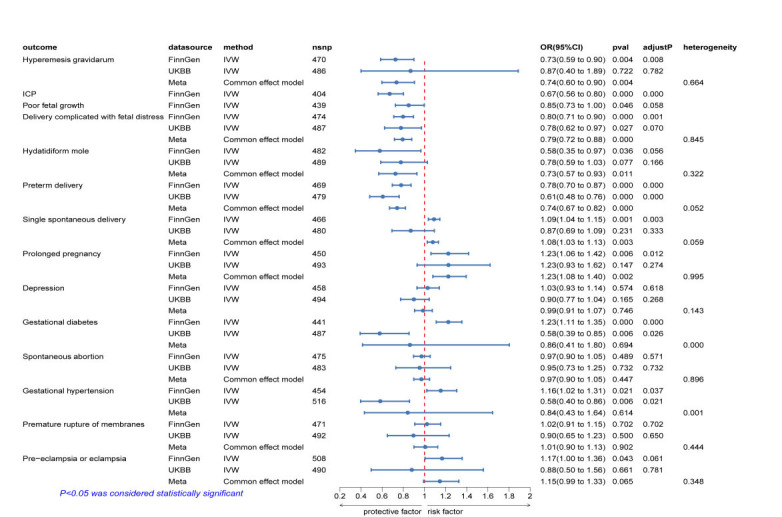
Meta-analysis to integrate the results from the UK Biobank and the FinnGen consortium. “OR” represents the effect of a standard deviation increase in genetically predicted BMR on the odds of each outcome. **Abbreviations:** CI, Confidence Interval; OR, Odds Ratio; ICP, Intrahepatic Cholestasis of Pregnancy; DCFD, Delivery Complicated with Fetal Distress; PD, Preterm Delivery; SSD, Single Spontaneous Delivery; PROP, prolonged pregnancy; GDM, gestational diabetes; GH, Gestational Hypertension.

This study employed the IVW approach to investigate the potential causal relationship between BMR and 14 obstetrical disorders. The outcomes of Cochran's Q test and MR-PRESSO indicated that no significant heterogeneity or multiplicity (*P*>0.05) were detected among the IVs of BMR and 14 obstetrical disorders (Table **2**). The two sample MR analysis indicated that BMR was negatively and causally associated with hyperemesis gravidarum (OR=0.730, 95%CI: 0.590-0.900, *P*=0.008), ICP (OR=0.670, 95%CI: 0.560-0.800, *P*<0.001), delivery complicated with fetal distress (OR=0.800, 95%CI: 0.710-0.900, *P*=0.001) and preterm delivery (OR=0.780, 95%CI: 0.700-0.870, *P*<0.001). For a 1-Standard Deviation (SD) increase in BMR, the risk of hyperemesis gravidarum, ICP, fetal distress, and preterm birth decreases by 27%, 33%, 20%, and 22%, respectively. BMR also exhibited a positive causal relationship with the risk of single spontaneous delivery (OR=1.090, 95%CI: 1.040-1.150, *P*=0.003), prolonged pregnancy (OR=1.230, 95%CI: 1.060-1.420, *P*=0.012), GDM (OR=1.230, 95%CI: 1.110-1.350, *P*<0.001) and gestational hypertension (OR=1.160, 95%CI: 1.020-1.310, *P*=0.037) (Supplementary Table **2** Fig. **1**). For a 1-SD increase in BMR, the risk of singleton natural delivery, Prolonged Pregnancy, GDM, and Gestational Hypertension increases by 9%, 23%, 23% and 16% respectively. The MR Steiger direction test revealed no inverse correlation between BMR and obstetrical disorders (Table **3**). A conclusive random-effects meta-analysis of the causality estimates from the two databases indicated that hyperemesis gravidarum (OR=0.740, 95%CI: 0.600-0.900, *P*=0.004), ICP (OR=0.670, 95%CI: 0.560-0.800, *P*<0.001), delivery complicated with fetal distress (OR=0.790, 95%CI: 0.720-0.880, *P*<0.001), single spontaneous delivery (OR=1.080, 95%CI: 1.030-1.130, *P*=0.003) and prolonged pregnancy (OR=1.230, 95%CI: 1.080-1.400, *P*=0.002). For a 1-SD increase in BMR, the risk of hyperemesis gravidarum, ICP, and fetal distress decreases by 26%, 33%, and 21%, respectively, while the risk of single spontaneous delivery and prolonged pregnancy increases by 8% and 23%. This finding is consistent with the above results (Fig. **2**).

### 3.3 Results of Multivariate MR Analysis

**Fig. (3):**
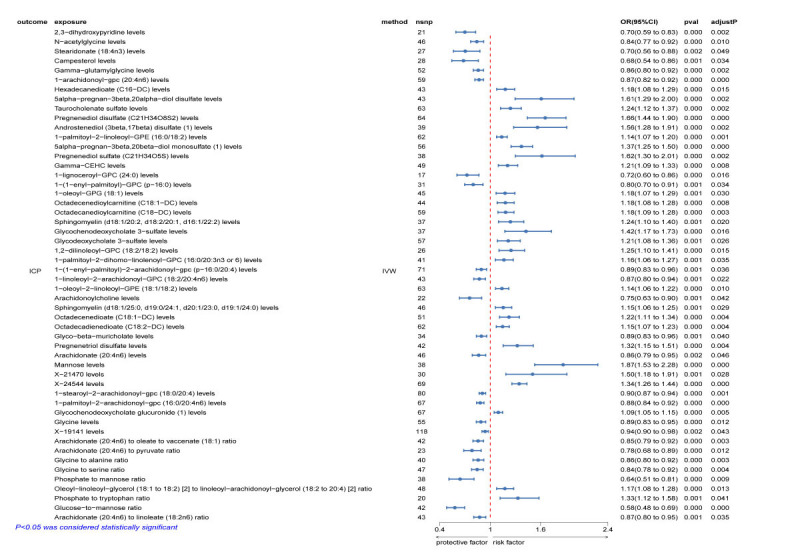
Mendelian randomization estimates of 1400 metabolites on the risk for ICP. “OR” represents the effect of a standard deviation increase in genetically predicted BMR on the odds of each outcome; **Abbreviations:** CI, Confidence Interval; OR, Odds Ratio; ICP, Intrahepatic Cholestasis of Pregnancy.

**Fig. (4):**
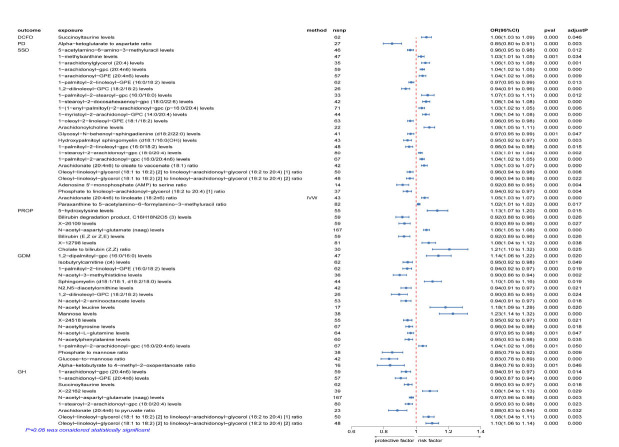
Mendelian randomization estimates of 1400 metabolites on the risk for six other obstetric diseases. “OR” represents the effect of a standard deviation increase in genetically predicted BMR on the odds of each outcome. **Abbreviations:** CI, Confidence Interval; OR, Odds Ratio; DCFD, Delivery Complicated with Fetal Distress; PD, Preterm Delivery; SSD, Single Spontaneous Delivery; PROP, prolonged pregnancy; GDM, gestational diabetes; GH, Gestational Hypertension.

**Fig. (5):**
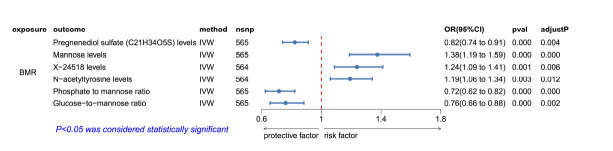
Mendelian randomization estimates of BMR on the positive metabolites. “OR” represents the effect of a standard deviation increase in genetically predicted BMR on the odds of each outcome. **Abbreviations:** CI, Confidence Interval; OR, Odds Ratio; BMR, Basal Metabolic Rate.

This study employed MVMR and mediation analysis to evaluate whether blood metabolites serve as mediators affecting the risk of BMR and obstetrical disorders. Initially, we assessed the potential correlation between 1400 blood metabolites and the eight obstetrical disorders causally associated with BMR. The findings revealed significant associations between specific metabolites and ICP, delivery complicated with fetal distress, single spontaneous delivery, prolonged pregnancy, GDM, and gestational hypertension, respectively (Supplementary Tables **3-7** Figs. **3** and **4**). We subsequently evaluated the potential correlation between BMR and blood metabolites linked to obstetrical disorders. We identified six metabolites (mannose levels, pregnenediol sulfate (C21H34O5S) levels, phosphate-to-mannose ratio, glucose-to-mannose ratio, X-24518 levels, and N-acetyltyrosine levels) that were causally associated with the risk of obstetrical disorders (Fig. **5**).

### 3.4 Results of Mediation Analysis

**Fig. (6):**
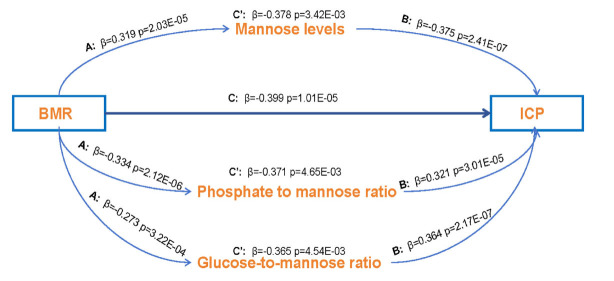
MVMR results and mediation effect calculation. “OR” represents the effect of a standard deviation increase in genetically predicted BMR on the odds of each outcome. **Abbreviations:** CI, Confidence Interval; OR, Odds Ratio; BMR, Basal Metabolic Rate.

Building on these causal associations, blood metabolites were used to evaluate the mediating role of basal metabolic rate and obstetric diseases. The findings indicated that the effect value (A) for the incidence of BMR at mannose levels was 0.319, while the effect value (B) for the incidence of mannose levels at ICP was -0.375, resulting in a mediator effect ratio of (A× B)/C' = 29.93%. The findings of this study indicate that BMR may significantly decrease the chance of developing ICP by partially enhancing mannose levels, with a mediation effect ratio of 29.93% (Fig. **6** Table **4** and Supplementary Table **8**).

## 4 DISCUSSION

The correlation between BMR and obstetrical disorders has garnered considerable attention. Nonetheless, the causal correlation between the two variables remains ambiguous. This study found that an elevation in BMR may decrease the likelihood of hyperemesis gravidarum, intrahepatic cholestasis of pregnancy, fetal distress, and preterm delivery, offering a novel perspective on the correlation between metabolic alterations and pregnancy complications. The mediation analysis further demonstrated that BMR may decrease the likelihood of ICP formation by positively influencing mannose levels. It suggests that mannose levels may not serve as an adequate mediator between BMR and the onset of ICP. Specific research suggests that the correlation between the two is predominantly affected by indirect mechanisms, such as hormonal fluctuations during pregnancy, improved nutritional health, and increased energy reserves [43, 44].

Maternal hormonal changes during pregnancy, particularly elevated levels of estrogen, progesterone, and human Chorionic Gonadotropin (hCG), critically regulate metabolism and fetal development. These hormones enhance nutrient absorption and utilization, ensuring fetal growth stability while reducing the risk of fetal distress [45]. Progesterone suppresses preterm labor by relaxing uterine muscles [46, 47], while estrogen optimizes placental blood flow for oxygen and nutrient delivery [48]. A moderately elevated BMR reflects hormonal equilibrium and metabolic adaptability, further supporting fetal health and minimizing adverse outcomes. Consequently, it is essential to maintain optimal nutritional conditions, monitor hormonal levels, and preserve adequate energy reserves to mitigate the risk of pregnancy-related issues.

We found that the protective effect of elevated BMR on pregnancy complications has significant practical significance. Per 1-SD increase in BMR, adjusted hazard ratios (95% CI) were 0.67 (0.61-0.73) for Intrahepatic Cholestasis of Pregnancy (ICP), 0.80 (0.75-0.85) for fetal distress, and 0.78 (0.72-0.84) for preterm birth. Furthermore, elevated BMR showed a significant association with increased likelihood of spontaneous vaginal delivery (OR=1.32, 95%CI 1.18-1.47). The increase in BMR reflects the optimization of metabolic homeostasis and the maintenance of sufficient energy reserves in pregnant women, which may enhance their adaptability to hormonal fluctuations during pregnancy by improving metabolic flexibility. Studies have shown that high BMR is associated with enhanced maternal metabolic activity. At the same time, adequate nutritional intake can maintain energy balance and improve immune function, indirectly alleviating symptoms of fatigue and discomfort [49]. Meanwhile, elevated BMR may improve fetal nutritional supply and reduce the risk of fetal distress by enhancing the efficiency of mitochondrial oxidative phosphorylation and promoting the uptake of glucose and fatty acids by the placenta. This process may involve the activation of the AMPK/mTOR signaling pathway, regulating angiogenesis [50] and nutrient transport [51]. Furthermore, an elevation in BMR typically coincides with an increase in blood volume and cardiac output during pregnancy. This change indicates that maternal metabolic adjustments are made to sustain placental function and fetal health. This metabolic response enhances blood flow to the placenta, supporting optimal placental function. An adequately functioning placenta is crucial for the baby to obtain nutrients and oxygen; therefore, optimal placental function supports normal fetal development and reduces the risk of fetal distress and preterm delivery [52]. This effect is consistent with the improvement effect of lifestyle interventions (such as moderate exercise) on metabolic rate in previous studies [47].

The current study additionally demonstrated the mediating effect of mannose levels on BMR and the probability of developing ICP. It also indicates that elevated mannose levels may enhance the efficacy of BMR in reducing the likelihood of developing ICP. Mannose is a monosaccharide primarily obtained from dietary sources and synthesized internally. Mannose orchestrates immune-metabolic crosstalk by suppressing glycolysis and activating the tricarboxylic acid (TCA) cycle through metabolic reprogramming, thereby elevating α-ketoglutarate (α-KG) to modulate Ten-Eleven Translocation (TET) enzyme-mediated DNA demethylation, which sustains T cell progenitor states and potentiates blockade efficacy in tumor models [53-55]. In addition, mannose protects the intestinal barrier by inhibiting TNF-α-mediated endoplasmic reticulum stress. It alleviates inflammation by regulating macrophage metabolism (such as reducing 3-phosphoglyceraldehyde) [56, 57], while improving insulin resistance and lipid metabolism disorders related to intestinal flora imbalance [58-60]. Animal studies have also shown that oral mannose can inhibit the expression of ketohexokinase in hepatocytes and alleviate liver steatosis and fibrosis [60-62].

Higher metabolic rates may enhance bile acid processing, reducing hepatic bile retention and ICP risk. Conversely, lower metabolism in pregnant women could impair liver function and bile acid metabolism, increasing ICP susceptibility. Current research focuses on ICP pathophysiology, fetal impacts, and prevention [63, 64], with limited data available on BMR changes. Although hepatic dysfunction and hormonal fluctuations in ICP patients may alter BMR, empirical validation is needed. Mannose, critical for hepatic processes such as gluconeogenesis and N-glycosylation, accumulates in liver dysfunction (*e.g.*, cirrhosis, NAFLD) and may serve as a biomarker [65-69, 19]. Its potential role in ICP pathophysiology and interaction with metabolic pathways (*e.g*., glycoprotein synthesis, glycolysis [70]) warrants investigation. While no studies directly link BMR to mannose levels, an elevated BMR may indirectly regulate mannose levels *via* increased metabolic demand, enhanced glucose utilization, or hormonal shifts [71, 72]. Although BMR alterations alone cannot predict pregnancy complications, their potential association with hypermetabolic states and mannose levels requires further validation.

### 4.1 Strengths of this Study

Firstly, the study employs Mendelian Randomization (MR) analysis, leveraging genetic variants as instrumental variables to infer causality while minimizing confounding biases inherent in observational studies. Multivariable MR and mediation analyses further enhance the validity of the findings by accounting for potential mediators and confounders. Secondly, this study is among the first to integrate MR and mediation analysis to explore the interplay between BMR, blood metabolites, and obstetrical outcomes, advancing the understanding of metabolic contributions to pregnancy complications. Finally, the findings provide actionable insights into metabolic pathways (*e.g.*, BMR and mannose) that can inform preventive strategies for obstetrical disorders, thereby bridging genetic epidemiology with clinical applications.

## 5 LIMITATIONS OF THIS STUDY

This study has several limitations. Firstly, MR analysis used genetic data from individuals of European ancestry, which limits its generalization to other races or geographical groups. Secondly, although we use the MR-intercept and MR-PRESSO global tests to identify and address pleomorphism in genetic variations, confounding variables (such as personality traits and mental conditions) between exposure and outcomes may still exist, which may lead to biased findings. Future investigations require prospective, multi-center cohorts with longitudinal metabolomic profiling and placental transcriptomics to dissect the causal pathways of BMR in pre-eclampsia and GDM, particularly through mitochondrial bioenergetics and immune-endocrine crosstalk. Randomized controlled trials are needed to evaluate whether the incidence or severity of pregnancy complications can be reduced by altering the basal metabolic rate. Mechanism research is also indispensable for clarifying the role of blood metabolites in regulating the relationship between BMR and pregnancy complications.

## CONCLUSIONS

This study suggests that higher Basal Metabolic Rate (BMR) may reduce risks of hyperemesis gravidarum, Intrahepatic Cholestasis of Pregnancy (ICP), fetal distress, and preterm delivery. Mannose levels were identified as a mediator between BMR and ICP risk. Understanding these causal relationships could inform targeted therapies, suggesting that maintaining BMR through moderate exercise during pregnancy may help prevent such complications.

## Figures and Tables

**Table 1:** The characteristics of data.

-	**Source**	**Cases**	**Controls**	**Sample Size**	**Population**
Exposure	-	-	-	-	-
BMR(ebi-a-GCST90029025)	UKBB	-	-	534,045	European
Outcome	-	-	-	-	-
Hyperemesis gravidarum	FinnGen R10	2677	195520	198197	European
	UKBB	240	227261	227501	European
Intrahepatic Cholestasis of Pregnancy	FinnGen R10	2784	227526	230310	European
/	UKBB	-	-	-	/
Poor fetal growth	FinnGen R10	4054	226256	230310	European
/	UKBB	-	-	-	/
Delivery complicated with fetal distress	FinnGen R10	9428	176810	186238	European
	UKBB	2267	219193	221460	European
Hydatidiform mole	FinnGen R10	425	162987	163412	European
	UKBB	1309	219979	221288	European
Preterm labour and delivery	FinnGen R10	9402	176810	186212	European
	UKBB	646	419885	420531	European
Single spontaneous delivery	FinnGen R10	117502	99373	216875	European
	UKBB	2041	418490	420531	European
Prolonged pregnancy	FinnGen R10	5158	182824	187982	European
	UKBB	1329	226172	227501	European
Postpartum depression	FinnGen R8	13657	236178	249835	European
	UKBB	5688	38595	44283	European
Gestational diabetes	FinnGen R10	14718	215592	230310	European
	UKBB	202	420329	420531	European
Spontaneous abortion	FinnGen R10	18680	162987	181667	European
	UKBB	1345	419186	420531	European
Gestational hypertension	FinnGen R10	9535	211957	221492	European
	UKBB	314	420217	420531	European
Premature rupture of membranes	FinnGen R10	8093	182824	190917	European
	UKBB	1080	419451	420531	European
Pre-eclampsia or eclampsia	FinnGen R10	7965	211852	219817	European
	UKBB	319	226285	226604	European

**Table 2:** Heterogeneity and pleiotropy test of the BMR IVs from 14 obstetric disease GWAS.

Exposure	Outcome	N.SNPs	Heterogeneity Test						Pleiotropy Test			MR-PRESSO *P*-value
			MR-Egger			Inverse Variance Weighted			MR-Egger			
			Q	Q_df	Q_*p*val	Q	Q_df	Q_*p*val	Intercept	se	*p*val	
BMR	Hyperemesis gravidarum	470	407.145	468	0.980	407.467	469	0.981	-0.002	0.004	0.571	0.989
	Intrahepatic cholestasis of pregnancy	404	222.869	402	1.000	224.456	403	1.000	0.005	0.004	0.208	1.000
	Poor fetal growth	439	294.413	437	1.000	294.461	438	1.000	-0.001	0.003	0.827	1.000
	Delivery complicated with fetal distress	474	387.660	472	0.998	387.663	473	0.998	0.000	0.002	0.952	0.998
	Hydatidiform mole	482	400.887	480	0.996	401.170	481	0.997	-0.005	0.009	0.595	0.996
	Preterm delivery	469	369.907	467	1.000	369.917	468	1.000	0.000	0.002	0.924	0.999
	Single spontaneous delivery	466	420.244	464	0.928	420.245	465	0.933	0.000	0.001	0.972	0.948
	Prolonged pregnancy	450	314.808	448	1.000	314.827	449	1.000	0.000	0.003	0.892	1.000
	Postpartum depression	458	388.690	456	0.990	388.802	457	0.991	-0.001	0.002	0.738	0.993
	Gestational diabetes	441	355.705	439	0.999	359.391	440	0.998	0.003	0.002	0.056	0.996
	Spontaneous abortion	475	368.480	473	1.000	368.589	474	1.000	0.000	0.001	0.742	1.000
	Gestational hypertension	454	402.295	452	0.955	406.079	453	0.945	0.004	0.002	0.052	0.896
	Premature rupture of membranes	471	356.317	469	1.000	356.715	470	1.000	0.001	0.002	0.528	1.000
	Pre-eclampsia or eclampsia	508	678.508	506	0.000	679.225	507	0.000	0.002	0.002	0.465	0.395

**Abbreviatons:** BMR, Basal Metabolic Rate; IVs, Instrumental Variables; GWAS, Genome-Wide Association Study; SNP, Single-Nucleotide Polymorphism; MR, Mendelian Randomization.

**Table 3:** Direction test from BMR to obstetrical diseases.

Exposure	Outcome	snp_r^2^.exposure	snp_r^2^.outcome	correct_causal_direction	steiger_*p*val
BMR	Hyperemesis gravidarum	0.072	0.002	TRUE	0
	Intrahepatic cholestasis of pregnancy	0.071	0.002	TRUE	0
	Poor fetal growth	0.071	0.002	TRUE	0
	Delivery complicated with fetal distress	0.072	0.002	TRUE	0
	Hydatidiform mole	0.075	0.002	TRUE	0
	Preterm delivery	0.071	0.002	TRUE	0
	Single spontaneous delivery	0.068	0.002	TRUE	0
	Prolonged pregnancy	0.071	0.002	TRUE	0
	Postpartum depression	0.07	0.002	TRUE	0
	Gestational diabetes	0.064	0.002	TRUE	0
	Spontaneous abortion	0.073	0.002	TRUE	0
	Gestational hypertension	0.066	0.002	TRUE	0
	Premature rupture of membranes	0.072	0.002	TRUE	0
	Pre-eclampsia or eclampsia	0.069	0.002	TRUE	0

**Abbreviation:** BMR, basal metabolic rate.

**Table 4:** The mediation effect of Basal metabolic rate on ICP *via* mediators.

Mediator	Total Effect C β(95%CI)	Direct Effect A β(95%CI)	Direct Effect B β(95%CI)	Mediation effect β	Mediated Proportion (%)
MR analysis BMR on ICP	-	-	-	-	-
Mannose levels	-0.399(-0.576, -0.222)	0.319(0.172, 0.465)	-0.375(-0.517, -0.232)	-0.119	29.93%
Phosphate to mannose ratio	-0.399(-0.576, -0.222)	-0.334(-0.471, -0.196)	0.321(0.170, 0.472)	-0.107	26.83%
Glucose-to-mannose ratio	-0.399(-0.576, -0.222)	-0.273(-0.422, -0.124)	0.364(0.227, 0.502)	-0.100	24.96%

**Note:** ‘Total effect’ indicates the effect of BMR on ICP, ‘direct effect A’ means the effect of BMR on mediators, ‘direct effect B’ shows the effects of mediators on ICP, and ‘mediation effect’ suggests the effect of BMR on ICP through mediators. Total effect, direct effect A, and direct effect IVW derived B; the mediation effect was derived by the coefficients method. All statistical tests were two-sided. *P*<0.05 was considered significant. **Abbreviations:** ICP, Intrahepatic Cholestasis of Pregnancy; MR, Mendelian Randomization; BMR, Basal Metabolic Rate

## Data Availability

The data was included in the main text or supplementary materials.

## LIST OF ABBREVIATIONS

BMR: Basal Metabolic Rate
GWAS: Genome-Wide Association Study
MR: Mendelian Randomization
MVMR: Multivariable Mendelian Randomization
IVW: Inverse Variance Weighted
ICP: Intrahepatic Cholestasis of Pregnancy
OR: Odds Ratio
CI: Confidence Interval
*P*: *P*-value
GDM: Gestational Diabetes Mellitus
MA: Mediation Analysis
STROBE-MR: Strengthening the Reporting of Observational Studies in Epidemiology Using Mendelian Randomization
IVs: Instrumental Variables
FinnGen: Finnish Genetic Study
UKBB: UK Biobank
SNPs: Single Nucleotide Polymorphisms
ldlink: Linkage Disequilibrium Link
FDR: False Discovery Rate
HCG: Human Chorionic Gonadotropin

## STANDARDS OF REPORTING

STROBE-MR guidelines were followed.
