# Supplementary material for: The Mediating Role of Blood Metabolites in the Association between Basal Metabolic Rate and Obstetrical Disorders: A Mendelian Randomization Analysis
Source: Endocr Metab Immune Disord Drug Targets. 2025 Jul 24;26:E18715303400445. doi: 10.2174/0118715303400445250718112316 (PMC13334263; doi:10.2174/0118715303400445250718112316)
Supplement: Supplementary file 1 [file EMIDDT-26-E18715303400445_SD1.pdf]

Supplementary Material

The Mediating Role of Blood Metabolites in the Association between Basal Metabolic Rate and Obstetrical Disorders: A Mendelian Randomization Analysis

Yanqiong Gan[1,2,#] , Xinlin Tan[2,#], Yu Tang[2], Qi Shi2 and Hongbo Qi[3,*]

[1]Department of Obstetrics, The First Affiliated Hospital of Chongqing Medical University, No.1, Youyi Road, Chongqing 400016, China; [2]Department of Obstetrics, The Affiliated Hospital of North Sichuan Medical College, No.63, Wenhua Road, Nanchong, 637000, China; [3]Department of Obstetrics, The First Affiliated Hospital of Chongqing Medical University, No.1, Youyi Road, Chongqing 400016; Chongqing Key Laboratory of Maternal and Fetal Medicine, Chongqing Medical University, Chongqing 400016, China; Joint International Research Laboratory of Reproduction and Development of Chinese Ministry of Education, Chongqing Medical University, 400016, China

STROBE-MR checklist of recommended items to address in reports of Mendelian randomization studies[1 2]

Item No.	Section	Checklist item	Page No.	Relevant text from manuscript
1	**TITLE and ABSTRACT**	Indicate Mendelian randomization (MR) as the study's design in the title and/or the abstract if that is a main purpose of the study	2	Methods: Utilizing summary data from GWAS, we conducted a univariate MR and multivariable MR (MVMR) analysis to evaluate the potential causal correlation between BMR and 14 obstetrical disorders.
	**INTRODUCTION**			
2	**Background**	Explain the scientific background and rationale for the reported study. What is the exposure? Is a potential causal relationship between exposure and outcome plausible? Justify why MR is a helpful method to address the study question	4	Recently, studies have established a correlation between BMR and certain obstetrical disorders.
3	**Objectives**	State specific objectives clearly, including pre-specified causal hypotheses (if any). State that MR is a method that, under specific assumptions, intends to estimate causal effects	4	Mendelian randomization (MR) is an analytical method that use genetic variations to deduce the bias between exposure factors and clinical outcomes. Genetic diversity is randomly allocated among individuals and established at birth, it could be unaffected by environmental and social variables.
	**METHODS**			
4	**Study design and data sources**	Present key elements of the study design early in the article. Consider including a table listing sources of data for all phases of the study. For each data source contributing to the analysis, describe the following:		

	a)	Setting: Describe the study design and the underlying population, if possible. Describe the setting, locations, and relevant dates, including periods of recruitment, exposure, follow-up, and data collection, when available.	5	Initially, we evaluated the instrumental variables (IVs) for BMR and blood metabolites. Subsequently, we examined the causal correlation between BMR and obstetrical disorders in the two databases (FinnGen and UKBB). Finally, we conducted a meta-analysis to integrate the results from the two databases. Furthermore, the MVMR analysis and mediation analysis were conducted to evaluate if blood metabolites served as potential mediators between BMR and obstetrical disorders.
	b)	Participants: Give the eligibility criteria, and the sources and methods of selection of participants. Report the sample size, and whether any power or sample size calculations were carried out prior to the main analysis	6	In this study, we limited the genetic origin of participants to Europeans to mitigate any potential bias arising from other ethnicities.
	c)	Describe measurement, quality control and selection of genetic variants	6	We detected single nucleotide polymorphisms (SNPs) strongly correlated (P<5.0×10-8), F-statistic greater than 10 and r^2<0.001 with BMR and blood metabolite expression as instrumental factors
	d)	For each exposure, outcome, and other relevant variables, describe methods of assessment and diagnostic criteria for diseases	6	Finally, we gathered GWAS summary-level data for 14 obstetrical disorders: hyperemesis gravidarum, hydatidiform mole, spontaneous abortion, premature rupture of membranes, poor fetal growth, delivery complicated with fetal distress, preterm delivery, gestational diabetes, gestational hypertension, pre-eclampsia or eclampsia, intrahepatic cholestasis of pregnancy, single spontaneous delivery, prolonged pregnancy, and postpartum depression. However, data concerning ICP and poor fetal growth were unavailable from the UK Biobank.
	e)	Provide details of ethics committee approval and participant informed consent, if relevant	5	The data concerning the pertinent phenotypes were sourced from published GWAS that have secured ethical approvals from their respective institutions. This paper utilizes solely publicly available data from these studies, obviating the necessity for further ethical approvals.
5	**Assumptions**	Explicitly state the three core IV assumptions for the main analysis (relevance, independence and exclusion restriction) as well assumptions for any additional or sensitivity analysis	6	The MR analysis was predicated on three fundamental assumptions:(1) The assumption of association: the instrumental variable exhibits a strong correlation with exposure, (2) the assumption of exclusivity: the instrumental variable is uncorrelated with confounders, and (3) the assumption of independence: the instrumental variable is not correlated with the result.
6	**Statistical methods: main analysis**	Describe statistical methods and statistics used		

	a)	Describe how quantitative variables were handled in the analyses (i.e., scale, units, model)	7	The study utilized R software version 4.3.1, with primary packages including “TwoSampleMR” (version 0.5.7) and “MRPRESSO” (version 1.0). The correlation between BMR and the probability of each obstetrical disorder was shown as odds ratios (ORs) alongside 95% confidence intervals (CIs), with differences deemed statistically significant at P<0.05.
	b)	Describe how genetic variants were handled in the analyses and, if applicable, how their weights were selected	6	We detected single nucleotide polymorphisms (SNPs) strongly correlated (P<5.0×10-8), F-statistic greater than 10 and r^2<0.001 with BMR and blood metabolite expression as instrumental factors.
	c)	Describe the MR estimator (e.g. two-stage least squares, Wald ratio) and related statistics. Detail the included covariates and, in case of two-sample MR, whether the same covariate set was used for adjustment in the two samples	7	In this study, we employed the Benjamini-Hochberg technique to compute the adjusted p-value and false discovery rate (FDR) in order to minimize false-positive outcomes.
	d)	Explain how missing data were addressed	6	Ultimately, considering the potential multidirectional effects of the genetic instrument on possible confounders, we excluded SNPs from this study's analysis by utilizing the ldlink database (https://ldlink.nih.gov/) to eliminate multidirectional SNPs linked to other potential confounders.
	e)	If applicable, indicate how multiple testing was addressed	/	/
7	**Assessment of assumptions**	Describe any methods or prior knowledge used to assess the assumptions or justify their validity	7	We primarily utilized IVW method to evaluate the causal connection between BMR and the 14 obstetrical disorders. The IVW results yielded the most precise analysis in the absence of horizontal pleiotrop. The random effects model was employed in the presence of heterogeneity, while a fixed effects model was utilized in its absence.
8	**Sensitivity analyses and additional analyses**	Describe any sensitivity analyses or additional analyses performed (e.g. comparison of effect estimates from different approaches, independent replication, bias analytic techniques, validation of instruments, simulations)	8	Sensitivity analysis comprises heterogeneity test, horizontal pleiotropy test, and reject-by-exclusion test. Initially, the Cochran's Q test was employed to identify the heterogeneity among all SNPs. Then horizontal pleiotropy and MR-Egger intercept test were used to detect the pleiotropic effect. We also utilized the MR-PRESSO program to eliminate any outliers detected in the MR analysis and re-evaluated the MR causality estimations.
9	**Software and pre-registration**			
	a)	Name statistical software and package(s), including version and settings used	7	The study utilized R software version 4.3.1, with primary packages including “TwoSampleMR” (version 0.5.7) and

				“MRPRESSO” (version 1.0).
	b)	State whether the study protocol and details were pre-registered (as well as when and where)	/	/
	**RESULTS**			
10	**Descriptive data**			
	a)	Report the numbers of individuals at each stage of included studies and reasons for exclusion. Consider use of a flow diagram	/	/
	b)	Report summary statistics for phenotypic exposure(s), outcome(s), and other relevant variables (e.g. means, SDs, proportions)		
	c)	If the data sources include meta-analyses of previous studies, provide the assessments of heterogeneity across these studies	8	The outcomes of Cochran's Q test and MR-PRESSO indicated that no significant heterogeneity or multiplicity (P>0.05) was detected among the IVs of BMR and 14 obstetrical disorders (Table 1).
	d)	For two-sample MR:<br>i. Provide justification of the similarity of the genetic variant-exposure associations between the exposure and outcome samples<br>ii. Provide information on the number of individuals who overlap between the exposure and outcome studies	/	/
11	**Main results**			
	a)	Report the associations between genetic variant and exposure, and between genetic variant and outcome, preferably on an interpretable scale	8	In the univariate MR analysis, we utilized the screening criteria of $r^2<0.001$, P<5×10-8 and an F-statistic greater than 10, this analysis ultimately identified 6603 IVs, all demonstrating robust associations with exposure. The pertinent parameters of the SNPs are outlined in Supplementary Table 1.
	b)	Report MR estimates of the relationship between exposure and outcome, and the measures of uncertainty from the MR analysis, on an interpretable scale, such as odds ratio or relative risk per SD difference	8	The univariable MR analysis indicated that BMR was negatively and causally associated with hyperemesis gravidarum (OR=0.730, 95%CI: 0.590~0.900, P=0.008), ICP (OR=0.670, 95%CI: 0.560~0.800, P<0.001), and delivery complicated with fetal distress (OR=0.800, 95%CI: 0.710~0.900, P=0.001), as well as with the risk of preterm delivery (OR=0.780, 95%CI: 0.700~0.870, P<0.001). BMR also exhibited a positive causal relationship with the risk of single spontaneous delivery (OR=1.090, 95%CI: 1.040~1.150, P=0.003), prolonged pregnancy (OR=1.230, 95%CI: 1.060~1.420, P=0.012), GDM (OR=1.230, 95%CI: 1.110~1.350, P<0.001), and gestational hypertension (OR=1.160, 95%CI: 1.020~1.310, P=0.037) .

	c)	If relevant, consider translating estimates of relative risk into absolute risk for a meaningful time period	/	/
	d)	Consider plots to visualize results (e.g. forest plot, scatterplot of associations between genetic variants and outcome versus between genetic variants and exposure)	9	A conclusive random-effects meta-analysis of the causality estimates from the two databases indicated that hyperemesis gravidarum (OR=0.740, 95%CI: 0.600~0.900, P=0.004), ICP (OR=0.670, 95%CI: 0.560~0.800, P<0.001), delivery complicated with fetal distress (OR=0.790, 95%CI: 0.720~0.880, P<0.001), single spontaneous delivery (OR=1.080, 95%CI: 1.030~1.130, P=0.003), and prolonged pregnancy (OR=1.230, 95%CI: 1.080~1.400, P=0.002) corroborated the aforementioned findings (Figure 3).
12	**Assessment of assumptions**			
	a)	Report the assessment of the validity of the assumptions	8	The outcomes of Cochran's Q test and MR-PRESSO indicated that no significant heterogeneity or multiplicity (P>0.05) was detected among the IVs of BMR and 14 obstetrical disorders (Table 1).
	b)	Report any additional statistics (e.g., assessments of heterogeneity across genetic variants, such as I^2, Q statistic or E-value)	/	/
13	**Sensitivity analyses and additional analyses**			
	a)	Report any sensitivity analyses to assess the robustness of the main results to violations of the assumptions	8	The outcomes of Cochran's Q test and MR-PRESSO indicated that no significant heterogeneity or multiplicity (P>0.05) was detected among the IVs of BMR and 14 obstetrical disorders (Table 1).
	b)	Report results from other sensitivity analyses or additional analyses		
	c)	Report any assessment of direction of causal relationship (e.g., bidirectional MR)	9	The MR Steiger direction test revealed no inverse correlation between BMR and obstetrical disorders (Table 2).
	d)	When relevant, report and compare with estimates from non-MR analyses	/	/
	e)	Consider additional plots to visualize results (e.g., leave-one-out analyses)	9	The findings revealed significant associations between certain metabolites and ICP, delivery complicated with fetal distress, single spontaneous delivery, prolonged pregnancy, GDM and gestational hypertension, respectively (Supplementary Tables 3-6, Figure 4).
	**DISCUSSION**			

14	**Key results**	Summarize key results with reference to study objectives	10	Nonetheless, the causal correlation between the two variables remains ambiguous. This study identified that elevated BMR may be a significant risk factor causally linked to certain obstetrical disorders, as demonstrated using univariate and multivariate MR analysis. The mediation analysis further demonstrated that BMR may decrease the likelihood of ICP formation by positively influencing mannose levels. It indicates that mannose levels may serve as an inadequate mediator between BMR and the onset of ICP.
15	**Limitations**	Discuss limitations of the study, taking into account the validity of the IV assumptions, other sources of potential bias, and imprecision. Discuss both direction and magnitude of any potential bias and any efforts to address them	13	This comprehensive investigation, which was conducted among a European population, establishes a basis for understanding a particular incident. Nevertheless, it is imperative to conduct further research to ascertain the generality of these findings and determine whether they hold true for diverse populations spanning the globe, especially in other countries. Currently, the GWAS statistics accessible in diverse databases tend to be broadly defined and lack fundamental demographic details like age brackets and gender breakdowns. Consequently, this poses challenges in conducting further subgroup evaluations, necessitating the compilation of GWAS data on a significantly larger scale for subsequent investigative endeavors.
16	**Interpretation**			
	a)	Meaning: Give a cautious overall interpretation of results in the context of their limitations and in comparison with other studies	14	The findings of this study indicate that BMR might decrease the likelihood of hyperemesis gravidarum, intrahepatic cholestasis of pregnancy, fetal distress, and preterm delivery. Moreover, we demonstrated the mediating effect of mannose levels on the relationship between BMR and the risk of developing ICP.
	b)	Mechanism: Discuss underlying biological mechanisms that could drive a potential causal relationship between the investigated exposure and the outcome, and whether the gene-environment equivalence assumption is reasonable. Use causal language carefully, clarifying that IV estimates may provide causal effects only under certain assumptions	11	Consequently, it is essential to sustain optimal nutritional condition, check hormonal levels, and maintain adequate energy reserves to mitigate the risk of pregnancy issues.
	c)	Clinical relevance: Discuss whether the results have clinical or public policy relevance, and to what extent they inform effect sizes of possible interventions	13	It is important to recognize that alterations in BMR, as a measure of overall metabolic health during pregnancy, don't directly determine pregnancy-related issues, necessitating further validation in future investigations.
17	**Generalizability**	Discuss the generalizability of the study results (a)	/	/

		to other populations, (b) across other exposure periods/timings, and (c) across other levels of exposure		
	**OTHER INFORMATION**			
18	**Funding**	Describe sources of funding and the role of funders in the present study and, if applicable, sources of funding for the databases and original study or studies on which the present study is based	15	This study was supported by Project approved by the Ministry of Industry and Information Technology of the People's Republic of China and the National Healthcare Commission for the 5G+Medical and Healthcare Application Pilot Project (Project Name: 5G+Maternal and Child Health Service and Management, JKZX2022-5G03).
19	**Data and data sharing**	Provide the data used to perform all analyses or report where and how the data can be accessed, and reference these sources in the article. Provide the statistical code needed to reproduce the results in the article, or report whether the code is publicly accessible and if so, where	15	We would like to thank the database for making GWAS data available to the public.
20	**Conflicts of Interest**	All authors should declare all potential conflicts of interest	15	Each author certifies that none of their personal or professional interests conflict.

This checklist is copyrighted by the Equator Network under the Creative Commons Attribution 3.0 Unported (CC BY 3.0) license.

1. Skrivankova VW, Richmond RC, Woolf BAR, Yarmolinsky J, Davies NM, Swanson SA, et al. Strengthening the Reporting of Observational Studies in Epidemiology using Mendelian Randomization (STROBE-MR) Statement. JAMA. 2021;under review.

2. Skrivankova VW, Richmond RC, Woolf BAR, Davies NM, Swanson SA, VanderWeele TJ, et al. Strengthening the Reporting of Observational Studies in Epidemiology using Mendelian Randomisation (STROBE-MR): Explanation and Elaboration. BMJ. 2021;375:n2233.

Supplememtary Table 2.

	Nsnp	Beta	Se	Pval	Lo_ci	Up_ci	or	Or_lci95	Or_upci95
BMR-ICP	404	-0.398910041	0.09033704	1.01E-05	-0.57597064	-0.221849442	0.671051066	0.56215895	0.801035957
BMR-ICP	504	-0.25792451	0.130655166	0.048371986	-0.514008635	-0.001840384	0.772653558	0.598093229	0.998161308
BMR-Pregnenediol sulfate (C21H34O5S) levels	565	-0.196084239	0.053000535	0.000215884	-0.299965288	-0.09220319	0.821942992	0.740843937	0.911919837
Pregnenediol sulfate	1	0.07748589	0.0858443	0.3667209	-0.090	0.245740813	1.080566994	0.913228628	1.278568141

(C21H34O5S) levels-ICP		8	44	53	769017				
Mediation effect		-0.01519376	0.01791388	0.3963512	-0.05368477	0.01799722	0.9849211	0.9477308	1.01816
BMR-ICP	404	-0.398910041	0.09033704	1.01E-05	-0.57597064	-0.221849442	0.671051066	0.56215895	0.801035957
BMR-ICP	505	-0.377922347	0.129112342	0.003421592	-0.630982538	-0.124862157	0.685283713	0.532068766	0.882618557
BMR-Mannose levels	565	0.318767629	0.074792671	2.03E-05	0.172173994	0.465361264	1.375431677	1.1878845	1.59258943
Mannose levels-ICP	1	-0.374605553	0.072528673	2.41E-07	-0.516761752	-0.232449353	0.687560431	0.596448873	0.79258989
Mediation effect		-0.1194121	0.03672799	0.001148963	-0.1984973	-0.05520977	0.887442	0.819962	0.9462866
BMR-ICP	404	-0.398910041	0.09033704	1.01E-05	-0.57597064	-0.221849442	0.671051066	0.56215895	0.801035957
BMR-ICP	505	-0.370576438	0.130941314	0.00465341	-0.627221414	-0.113931462	0.69033628	0.534073711	0.892319111
BMR-Phosphate to mannose ratio	565	-0.333537288	0.070339234	2.12E-06	-0.471402187	-0.195672389	0.716385186	0.624126513	0.822281579
Phosphate to mannose ratio-ICP	1	0.320864982	0.076900484	3.01E-05	0.170140033	0.47158993	1.37831947	1.185470845	1.602540096
Mediation effect		-0.1070204	0.0345907	0.001975396	-0.1819197	-0.04707484	0.8985073	0.8336683	0.954016
BMR-ICP	404	-0.398910041	0.09033704	1.01E-05	-0.57597064	-0.221849442	0.671051066	0.56215895	0.801035957
BMR-ICP	505	-0.365131628	0.128654055	0.004538456	-0.617293577	-0.11296968	0.694105281	0.539402315	0.893177741
BMR-Glucose-to-mannose ratio	565	-0.273285213	0.075977399	0.000321998	-0.422200915	-0.124369511	0.760875745	0.655602306	0.883053483

Glucose-to-mannose ratio-ICP	1	0.36437081	0.070281253	2.17E-07	0.226619554	0.502122066	1.439607936	1.254352564	1.652223681
Mediation effect		-0.09957715	0.0341147 5	0.003512819	-0.1731451	-0.03999667	0.9052201	0.8410156	0.9607926
BMR-GDM	441	0.204069607	0.050752775	5.80E-05	0.104594168	0.303545045	1.226383515	1.11025994	1.354652611
BMR-GDM	505	0.102724409	0.069447757	0.139097312	-0.033393196	0.238842013	1.108185961	0.967158202	1.269777913
BMR-Mannose levels	565	0.318767629	0.074792671	2.03E-05	0.172173994	0.465361264	1.375431677	1.1878845	1.59258943
Mannose levels-GDM	1	-0.17693218	0.038877905	5.34E-06	-0.253132874	-0.100731487	0.837836605	0.776364716	0.904175784
Mediation effect		-0.05640025	0.0183619 3	0.002129247	-0.09622087	-0.02465924	0.9451608	0.9082634	0.9756423
BMR-GDM	441	0.204069607	0.050752775	5.80E-05	0.104594168	0.303545045	1.226383515	1.11025994	1.354652611
BMR-GDM	501	0.157909086	0.070594252	0.025295836	0.019544352	0.29627382	1.171059725	1.019736594	1.34483835
BMR-X-24518 levels	564	0.214165409	0.066542222	0.001288679	0.083742654	0.344588164	1.238827551	1.087349033	1.411408531
X-24518 levels-GDM	2	0.022626026	0.026417817	0.391738729	-0.029152895	0.074404946	1.022883936	0.971267951	1.077242944
Mediation effect		0.004845712	0.006112897	0.4279509	-0.006376491	0.01819353	1.004857	0.9936438	1.01836
BMR-GDM	441	0.204069607	0.050752775	5.80E-05	0.104594168	0.303545045	1.226383515	1.11025994	1.354652611
BMR-GDM	503	0.161886951	0.069975108	0.020695528	0.024735739	0.299038164	1.175727319	1.025044205	1.348561089
BMR-N-acetyltyrosine levels	564	0.175777088	0.060094239	0.003444297	0.05799238	0.293561796	1.19217228	1.05970692	1.341196058
N-acetyltyrosine levels-GDM	1	0.017794016	0.024317893	0.464336297	-0.029869054	0.065457086	1.017953273	0.970572618	1.06764692

Mediation effect		0.00312778	0.004642265	0.5004629	-0.005463154	0.0133224	1.003133	0.9945517	1.013412
BMR-GDM	441	0.204069607	0.050752775	5.80E-05	0.104594168	0.303545045	1.226383515	1.11025994	1.354652611
BMR-GDM	505	0.097467167	0.069962376	0.1635784	-0.03965909	0.234593425	1.102375247	0.961117037	1.264394593
BMR-Phosphate to mannose ratio	565	-0.333537288	0.070339234	2.12E-06	-0.471402187	-0.195672389	0.716385186	0.624126513	0.822281579
Phosphate to mannose ratio-GDM	1	0.176420579	0.040960092	1.65E-05	0.0961388	0.256702359	1.192939679	1.100911859	1.292660321
Mediation effect		-0.05884284	0.01867977	0.001632237	-0.09925272	-0.026428	0.9428549	0.9055138	0.9739182
BMR-GDM	441	0.204069607	0.050752775	5.80E-05	0.104594168	0.303545045	1.226383515	1.11025994	1.354652611
BMR-GDM	505	0.105751925	0.069012534	0.125434057	-0.029512642	0.241016491	1.111546095	0.970918604	1.272542021
BMR-Glucose-to-mannose ratio	565	-0.273285213	0.075977399	0.000321998	-0.422200915	-0.124369511	0.760875745	0.655602306	0.883053483
Glucose-to-mannose ratio-GDM	1	0.182663458	0.037546751	1.14E-06	0.109071827	0.25625509	1.200410352	1.115242451	1.292082283
Mediation effect		-0.04991922	0.01749379	0.004323516	-0.0878335	-0.01961473	0.9513063	0.9159134	0.9805764
